# Evaluation of *Viburnum opulus* L. Fruit Phenolics Cytoprotective Potential on Insulinoma MIN6 Cells Relevant for Diabetes Mellitus and Obesity

**DOI:** 10.3390/antiox9050433

**Published:** 2020-05-16

**Authors:** Małgorzata Zakłos-Szyda, Agnieszka Kowalska-Baron, Nina Pietrzyk, Anna Drzazga, Anna Podsędek

**Affiliations:** 1 Institute of Molecular and Industrial Biotechnology, Department of Biotechnology and Food Sciences, Lodz University of Technology, Stefanowskiego 4/10, 90-924 Lodz, Poland; nina.pietrzyk@dokt.p.lodz.pl (N.P.); anna.drzazga@p.lodz.pl (A.D.); anna.podsedek@p.lodz.pl (A.P.); 2 Institute of Natural Raw Materials and Cosmetics, Department of Biotechnology and Food Sciences, Lodz University of Technology, Stefanowskiego 4/10, 90-924 Lodz, Poland; agnieszka.kowalska-baron@p.lodz.pl

**Keywords:** *Viburnum opulus*, phenolic compounds, cytoprotection, pancreatic β-cells, insulin secretion

## Abstract

In this study, the influence of guelder rose (*Viburnum opulus*) fruit fresh juice (FJ) and a phenolic-rich fraction (PRF) isolated from juice on mice insulinoma MIN6 cells activities was investigated. Extracts were able to decrease intracellular oxidative stress at the highest non-cytotoxic concentrations. They induced glucagon-like peptide-1 (GLP-1) secretion in the presence of an elevated glucose concentration, and they inhibited in vitro activity of the dipeptidyl peptidase-4 (DPP4) enzyme. Nonetheless, inhibition of glucose-stimulated insulin secretion was detected, which was accompanied by a decrease of cellular membrane fluidity and hyperpolarization effect. In addition, the increase of free fatty acid uptake and accumulation of lipid droplets in MIN6 cells were observed. Elevated extract concentrations induced cell apoptosis through the intrinsic mitochondrial pathway with activation of initiatory caspase-9 and downstream caspases-3/7. The fluorescence-quenching studies indicated that PRF extract has binding affinity to human serum albumin, which is one of the factors determining drug bioavailability. Taken together, despite the cytoprotective activity against generated intracellular oxidative stress, *V. opulus* revealed potential toxic effects as well as decreased insulin secretion from MIN6 cells. These findings are relevant in understanding *V. opulus* limitations in developing diet supplements designed for the prevention and treatment of postprandial glucose elevation.

## 1. Introduction

In recent years, increased attention has been paid to research on phytocompounds with health beneficial properties, which can be found in lesser known horticultural plants. Among unpopular plants in Poland, although very often used as components of the human diet in Eastern Europe and Turkey, is *Viburnum opulus* L. This flowering plant is named guelder rose, snowball tree, European cranberry bush rose, or cramp bark [1,2]. Its rounded, red, and edible fruit, although bitter and astringent, can be found in food products such as juice, jams, jellies, marmalades, and fermented drinks. Its fruit, bark, leaves, and flowers have also been applied in folk medicine for the treatment of menstrual, stomach, and kidney cramps, duodenal ulcers, high blood pressure, heart troubles, coughs, colds, and neurosis [3]. Their activity is due to the presence of many different phytocompounds such as phenolics, triterpenoids, saponins, glycosides, iridoids, and vitamins [4]. Taking into account the amounts of phenolic compounds, the most noteworthy are fruit with a total content of 400 to 1460 mg/100 g fresh fruit [1]. The phenolic compound composition of *V. opulus* fruit was described previously and includes chlorogenic acid, flavanols, and procyanidins [5,6,7]. Whereas the antioxidant potential of guelder rose constituents is well characterized, its biological activity on the cellular model is not known very well. There are few studies revealing its anticancer properties against different cell lines, yet they match cytotoxicity with down-regulation of the cellular antioxidant defense system, mitochondria collapse, and cellular death induction [4,8,9,10,11,12]. Furthermore, even less data indicate the cytoprotective activity of *V. opulus*, especially in regard to diet-related chronic diseases generated by increased oxidative stress [13,14]. Indeed, in obese humans, elevated levels of free fatty acids, glucose, and inflammation lead to insulin resistance [15,16]. To maintain plasma glucose at normal levels and regulate glucose uptake by peripheral tissues, pancreatic β-cells must adjust their function to compensate for insulin resistance, which in turn potentiates intracellular oxidative stress [16]. Moreover, due to the low level of antioxidant protection, β-cells suffer from metabolic impairment, and glucose-stimulated insulin secretion is attenuated [17]. However, glucose-stimulated insulin secretion (GSIS) is regulated also by other factors, such as incretin hormones secreted in the gut such as glucagon-like peptide-1 (GLP-1). After GLP-1 binds to its receptor, GLP1R, potentiation of insulin secretion from β-cells occurs [18]. Yet, GLP-1 activity is influenced by its fast degradation catalyzed by dipeptidyl peptidase-4 (DPP4); thus, administration of DPP4 inhibitor increases GLP-1 biological activity, enhancing insulin secretion and improving glucose cellular uptake [18,19]. 

Because intake of *V. opulus* fruit may increase the antioxidant capacity of the body, and consequently counteract oxidative stress, we decided to investigate its influence on the prevention of obesity and type 2 diabetes. Our previous studies have identified potent antidiabetic activities of guelder rose as the inhibitor against α-amylase, α-glucosidase, and protein tyrosine phosphatase 1B (PTP1B) [20]. Furthermore, the phenolic-rich fraction (PRF) decreased free fatty acids and glucose uptake, as well as accumulation of lipid droplets in Caco-2 cells, revealing potential anti-obesity properties [5]. Taking into account that the pancreas is involved in nutrient metabolism regulation and glucose homeostasis, we wanted to determine the influence of *V. opulus* on β-cells. We previously found pancreatic βTC3 cells to have low level of antioxidant protection, which was supported by guelder rose phenolics activity [20]. Here, the mouse insulinoma MIN6 cell line was selected as the cellular model, which displays characteristics of pancreatic β-cells insulin secretion in response to glucose and other secretagogues [21,22]. As a source of biologically active phenolic compounds, fresh juice (FJ) and the PRF obtained from guelder rose juice were used. The identified phenolic compounds and their quantities were described in detail previously [5], and chemical characteristics are briefly presented in Table 1. The phenolics content in FJ reached a value of 10.32 mg/g in preparation, but sugars, proteins, organic acids, and other mineral ingredients were also present. Purification of juice performed via solid-phase extraction on a Sep-Pac C18 column allowed us to obtain the PRF, where phenolics reached 827.00 mg/g in preparation. As data demonstrated (Table 1), the juice purification process resulted in an 80-fold increase in the concentration of phenolic compounds. In the tested samples there were 10 major phenolics detected. As the main phenolic compound in both extracts, the chlorogenic acid amount in FJ was equal to 8 mg/g in preparation, whereas in PRF it reached 645 mg/g. Quantitatively, flavanols were the second most prominent component of the preparations with (+)-catechin as the main chemical. Both extracts also contained procyanidins B1 and B2. Among anthocyanins, different cyanidin glycosides have been identified with cyanidin 3-sambubioside as the main pigment. Flavonols occurred at the lowest concentration in the extracts. Due to low concentrations, neochlorogenic acid and quercetin were detected only in the PRF.

The purpose of this study was to determine the potential cytoprotective effect of *V. opulus* phenolic extracts against oxidative stress chemically induced by a potent pro-oxidant, *tert*-butylhydroperoxide (*t*-BOOH), as well as hypoglycemic properties involving influences on GSIS, GLP-1 secretion, DPP4 inhibition, and free fatty acid uptake. Mechanisms of extract cytotoxicity, type of cellular death induced, membrane effects, and extract components binding to human serum albumin were evaluated. 

## 2. Materials and Methods 

### 2.1. Chemicals and Reagents

All chemicals used, if not stated otherwise, were obtained from Sigma-Aldrich (Steinheim, Germany). Ultrapure water (Simplicity^®^ Water Purification System, Millipore, Marlborough, MA, USA) was used to prepare all solutions. 

### 2.2. Preparation of V. opulus Extracts

As a plant material fresh *Viburnum opulus* L. fruit were used (account number 18162), which were obtained from Rogów Arboretum, Warsaw University of Life Sciences (Rogów, Poland). After fruit homogenization and centrifuging (5000 rpm for 10 min) FJ was obtained. FJ purification by solid-phase extraction with C-18 Sep-Pak cartridge (10 g capacity, Waters Corp., Milford, MA, USA; 12-Port Vacuum Manifold system) and methanolic elution processes allowed to isolate PRF. To perform biological activity assays a stock solution of PRF at concentration 100 mg/mL in 50% dimethyl sulfoxide (DMSO) was prepared. Identified phenolic compounds and their quantities were described previously with details [5]. 

### 2.3. Cell Culture and Exposure Conditions

The murine-adherent insulinoma MIN6 cells were kindly provided by Dr Jun-ichi Miyazaki from the Division of Stem Cell Regulation Research, Osaka University, Japan [22]. Min6 cells were grown in Dulbecco′s Modified Eagle′s Medium (DMEM) medium with high glucose supplemented with 10% fetal bovine serum (FBS) supplemented with 50 µM β-mercaptoethanol, 100 U/mL penicillin, 100 µg/mL streptomycin, and 25 µg/mL amphotericin. Tested extracts were dissolved in 50% DMSO at a concentration of 100 mg/mL and were further diluted with culture medium [5]. The extract concentrations used in biological studies are presented in the descriptions of the tests carried out. All cell culture experiments were performed in a humidified 5% CO_2_ and 95% atmosphere at 37 °C. 

All cell culture reagents were obtained from Life Technologies (Carlsbad, CA, USA). Microscopic observations were performed using the fluorescent microscope Nikon TS100 Eclipse (Tokyo, Japan) under 200× magnification, if not stated otherwise. All the experimental measurements, if not stated otherwise, were performed using the Synergy 2 BioTek Microplate Reader (Winooski, VT, USA).

### 2.4. Cell Viability

For biological studies MIN6 cells were seeded into 96-well plates (10^4^ cells per well) in 100 µL of culture medium and grown for 24 h. Next, cells were incubated overnight in the presence of the studied extracts diluted in culture medium. Metabolic activity was evaluated with fluorescent measurement of PrestoBlue reagent at F530/590 nm. Values obtained were used to calculate cell metabolic activity expressed as the percentage of the response of the control cells (cells treated with equal volume of the vehicle instead of the preparation). As the vehicle corresponding volume of medium with 50% DMSO mixture was added, the final concentration of DMSO in the cell culture medium did not exceed 0.1%. To study cytoprotective properties of extracts, after cells 24 h pre-incubation with extracts at IC_0_ concentrations 500 μM *t*-BOOH was added for 2 h, and cellular viability with PrestoBlue assay was studied [23]. 

### 2.5. Detection of Intracellular Reactive Oxygen Species Generation 

The effect of extracts on intracellular generation of reactive oxygen species (ROS) was checked with dichloro-dihydro-fluorescein diacetate (DCFH-DA) dye. After 24 h treatment with the extracts, the medium was changed into phosphate buffered saline (PBS), DCFH-DA (10 μM) was added for 30 min and fluorescence at F485/530 nm was measured. As the positive control 500 µM *t*-BOOH was used. To study cytoprotective properties of extracts, after cells 24 h pre-incubation with extracts at IC_0_ concentrations 500 μM *t*-BOOH was added for 2 h, and the intracellular level of ROS was measured.

### 2.6. Measurement of Glutathione Peroxidase Activity and H_2_O_2_ Level

The effect of extracts on the activity of glutathione peroxidase was studied with a Glutathione Peroxidase Assay (GPX) kit (Cayman Chemical, Ann Arbor, MI, USA). Briefly, nicotinamide adenine dinucleotide phosphate (NADPH) and cumene hydroperoxide were added to the cell lysate mixture of co-substrate, and absorbance at 340 nm was measured. Level of hydrogen peroxide was measured with Hydrogen Peroxide Colorimetric Assay (Sigma-Aldrich) according to the manufacturer’s instructions. The absorbance measurement at 540 nm of the level of ferrous oxidation with Xylenol Orange (FOX) agent in the presence of sorbitol was performed. 

### 2.7. Measurement of ATP Production

After MIN6 cell incubation with the extracts for 24 h single reagent from CellTiter-Glo^®^ Luminescent Cell Viability Assay (Promega Corporation, Madison, WI, USA) was added to the cells, then the luminescent signal was measured.

### 2.8. Measurement of Mitochondrial Membrane Potential (MMP)

The mitochondrial membrane potential (MMP) was assayed with a 5,5′,6,6′-Tetrachloro-1,1′,3,3′-tetraethyl-imidacarbocyanine iodide (JC-1) probe. After 24 h treatment with the extracts, the medium was changed, and JC-1 (1 μg/mL) was added for 20 min [23]. Then, the cells were washed with serum-free medium, and fluorescent signals at F485/530 and F530/620 nm were measured.

### 2.9. Phosphatidylserine Externalization and Membrane Permeabilization

The Annexin-V-FITC assay kit (Cayman Chemical, Ann Arbor, MI, USA) was used to quantify the level of externalized phosphatidylserine (PS) of apoptotic cells. After treatment with extracts, cells were washed with phosphate buffer solution and incubated for 10 min with 0.25 μg/mL Annexin-V-FITC (fluorescein isothiocyanate). The level of PS was measured by the change in fluorescence at F485/530 nm. Simultaneously, the membrane permeabilization was measured with 1 µg/mL propidium iodide (PI) (fluorescent signal at F535/620 nm).

### 2.10. Detection of Caspases 3/7 and 9 Activities

The late stage of apoptosis was measured by an Apo-ONE^®^ Homogeneous Caspase-3/7 Assay according to the manufacturer’s instructions (Promega Corporation). After 24 h of treatment, cells were lysed, the assay buffer with Z-DEVD-R110 substrate was added, and the fluorescence was measured after 30 min (excitation/emission = 485/530 nm). Activation of caspase-9 was measured by a Caspase-Glo^®^ 9 Assay according to the manufacturer’s instructions (Promega Corporation). After 24 h treatment, a luminogenic substrate containing the LEHD sequence selective for caspase-9 was added to the cells, and luminescence was measured after 45 min. Chromatin condensation was assessed by 4’,6-diamidino-2-phenylindole (DAPI) staining. Briefly, after incubation, cells were fixed with 1% paraformaldehyde solution in PBS, stained with 1 μg/mL DAPI in the dark for 30 min, and visualized under a fluorescence microscope. 

### 2.11. Evaluation of Membrane Fluidity and Lateral Phase Separation

The level of cell membrane fluidity was investigated with Laurdan labeling. Briefly, after 24 h of treatment, cells were incubated with 2 µM Laurdan for 30 min, and the fluorescence signals at F360/440 and F360/490 were measured [24,25]. The fluorescence emission spectra of Laurdan-labeled cell suspensions (before and after extract component addition) were recorded using a FluoroMax4 (Jobin Yvon Spex) spectrofluorometer (Horiba Scientific, Piscataway, NJ, USA). All measurements were performed in a standard quartz cuvette at 20 °C under gentle stirring conditions. From the obtained fluorescence intensity data, the Generalized Polarization (GP) was calculated according to the formula:

(1) $$\mathrm{GP}=\frac{I_{440}-I_{490}}{I_{440}+{\;I}_{490}}$$

where I_440_ is fluorescence intensity at 440 nm, and I_490_ is fluorescence intensity at 490 nm. 

To check the influence of preparations on lateral phase separation after cell incubation with extracts, they were labeled with 2 μM pyrene for 20 min. Then, the fluorescence emission spectra were registered with a FluoroMax 4 (Jobin Yvon Spex) spectrofluorometer using an excitation wavelength set to 339 nm. Results were expressed as the ratio between the excimer (396 nm) and monomer fluorescence (470 nm).

### 2.12. Transmembrane Potential

After cell labeling with 2 µM DiBAC_4_(3), the fluorescence signal (F485/530 nm) was measured before and after cell incubation with compounds for 30 min. The transmembrane potential changes were evaluated according to the following equation:

membrane potential [%] = (I_0_ − I_30_)/I_0_ × 100 (2)

where I_0_ is fluorescence intensity at time 0 min, and I_30_ is fluorescence intensity after 30 min. 

### 2.13. Insulin Secretion 

Cells were seeded on 24-well plates and cultured 48 h before the experiment. Then, they were pre-incubated for 1 h with buffer (25 mM HEPES, 125 mM NaCl, 6 mM KCl, 1.2 mM MgCl_2_:6H_2_O, 1.3 mM CaCl_2_·2H_2_O, 2 mM glucose; pH 7.4). Subsequently, cells were incubated with extracts for 1 h, and buffer samples were collected. Next, the same cells were incubated for another 1 h with fresh buffer containing 20 mM glucose tested extracts, and buffer samples were collected. In cell lysates obtained with 0.1% Triton X-100 with PBS, the protein level was quantified with a Bradford assay. The insulin secreted in buffers was measured with a Mercodia Mouse Insulin ELISA kit (Mercodia AB, Uppsala, Sweden) according to the procedure. Insulin levels were normalized to protein content.

### 2.14. GLP-1 Secretion

The murine GLUTag cells were kindly provided by Prof F. Urbano (University of Catania, Catania, Italy) with permission from Prof D.J. Drucker (University of Toronto, Toronto, ON, Canada) [26]. Cells were cultured in DMEM medium with 5.6 mM glucose, supplemented with 10% FBS, 100 U/mL penicillin, and 100 µg/mL streptomycin. For the GLP-1 secretion experiments, the cells were plated in six-well plates and cultured for 72 h in standard culture medium. Prior to stimulation with extracts, the cells were pre-incubated for 2 h in either low-glucose or high-glucose DMEM (5.5 or 25 mM glucose respectively). Then, the medium was changed to a fresh portion supplemented with either FJ or PRF, and the cells were incubated for 2 h. Next, the culture media samples were collected to measure the GLP-1 concentration via ELISA (BlueGene). For the means of normalization, the cells were lysed with 0.1 M HCl with repetitive freezing and thawing, and total protein content was measured via the Bradford method.

### 2.15. Dipeptidyl Peptidase-4 (DPP4) Inhibition

The influence of extracts on dipeptidyl peptidase-4 activity was determined with a DPP4 Inhibitor Screening Kit (Sigma-Aldrich) according to the manufacturer’s protocol. Release of fluorescent product from non-fluorescent substrate was detected at 0 min and after 30 min incubation at F360/460 nm. The inhibitory potential of the extract is presented in relative inhibition [%] units. 

### 2.16. Nile Red Staining

After MIN6 cells incubation in FBS-free medium with extracts in the presence of 100 µM oleic acid for 24 h, cells were washed with cold PBS and fixed in 5% paraformaldehyde for 30 min. After staining with 1 µg/mL Nile red solution for 40 min, the fluorescent signal at F485/530 was measured.

### 2.17. Fatty Acid Uptake

Measurement of fatty acid uptake by cells was performed with Fatty Acid Uptake Kit, Sigma-Aldrich. Briefly, after MIN6 cells incubation with extracts, to FBS- and glucose-free medium fluorescent probe TF2-C12 was added for 1 h, and the fluorescent signal at F485/530 nm was measured.

### 2.18. Human Serum Albumin (HSA) Fluorescence Quenching

The fluorescence spectra of 15 µM HSA in Tris buffer pH 7.4 before and after adding increasing concentrations of PRF were recorded using the excitation wavelength set at 295 nm with a FluoroMax 4 (Jobin Yvon Spex) spectrofluorometer. All measurements were performed in a standard quartz cuvette at 20 °C. The time-resolved fluorescence of HSA before and after adding increasing concentrations of PRF was measured using the FL900CDT time-correlated single-photon counting fluorimeter from Edinburgh Analytical Instruments (Edinburgh, UK). The excitation and emission wavelengths were set to 295 and 350 nm, respectively. Data acquisition and analysis of the obtained results were performed with the use of software provided by Edinburgh Analytical Instrumentation. The time-resolved measurements were performed at 20 °C in a standard quartz cuvette.

### 2.19. Statistical Analysis

Results are presented as means of at least three independent experiments ± standard deviations. Differences between data groups were All calculations were evaluated for significance using one-way ANOVA followed by Dunnett’s test with GraphPad Prism 6.0 software (GraphPad Software, Inc., La Jolla, CA, USA). *p* ≤ 0.05 was considered statistically significant. 

## 3. Results and Discussion

### 3.1. V. opulus Phenolics Influence on Cellular Viability

**Figure 1:**
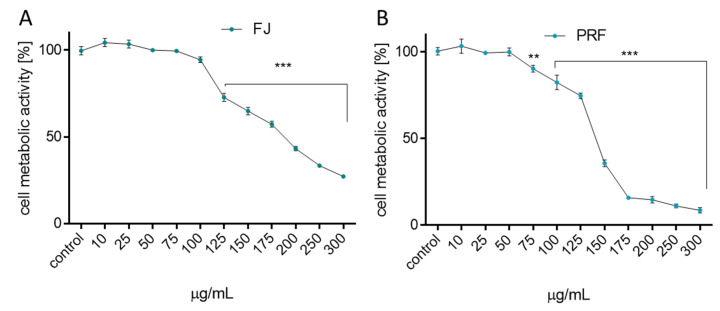
The influence of *V. opulus* extracts on MIN6 cell metabolic activity determined by PrestoBlue assay after 24 h exposure of juice (FJ) (**A**) and PRF (**B**). Control cells were not exposed to any compound but the vehicle; values are means ± standard deviations from at least three independent experiments, *n* ≥ 12; statistical significance was calculated versus control cells (untreated), ** *p* ≤ 0.01, *** *p* ≤ 0.001.

**Figure 2:**
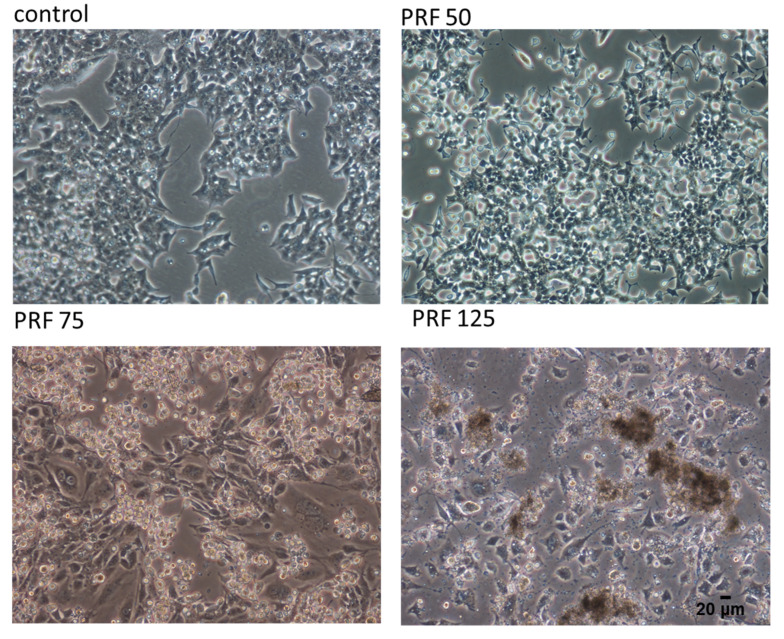
The influence of *V. opulus* PRF extract on the morphology of MIN6 cells treated for 24 h; control cells were not exposed to any compound but the vehicle; randomly chosen fields were photographed at × 200.

First, the influence of extracts on MIN6 cells metabolic activity was checked. Cells were incubated for 24 h in the presence of extract concentrations from 10 to 300 µg/mL (µg of freeze-dried extract/mL). As presented in Figure 1A,B the metabolic activity decreased with increasing extract concentrations starting from 50 µg/mL. The highest cytotoxicity was revealed with PRF, where 175 µg/mL dosage decreased cellular activity to almost 10% compared to the control cells. FJ had a weaker influence on cellular viability: the same dosage inhibited metabolic activity by 45%, whereas the highest concentration tested (300 µg/mL) decreased cell viability to 20%. The obtained IC_50_ value, thus the concentration of a certain compound required to reduce the cell metabolic activity level to 50% of the control, confirmed the observed higher cytotoxicity of PRF extract with IC_50_ = 135 µg/mL (respectively, for FJ IC_50_ = 180 µg/mL). The highest non-cytotoxic concentrations (IC_0_) selected for further studies were 75 µg/mL for juice and 50 µg/mL for the polyphenolic fraction, respectively. Additionally, the morphological changes exhibited by MIN6 cells were evaluated to determine whether the PRF extract is involved in development of abnormal cell morphology. As registered in Figure 2, cells cultured under normal conditions, thus not exposed to any compound, appeared polygonal in shape and grew closely attached to other cells. After PRF treatment at IC_0_, the cells had more irregular shapes with pointed protrusions. Treatment with higher PRF concentrations decreased the number of adherent cells, and the cells became loosely attached and rounded (75 µg/mL), whereas incubation with a high dose of PRF (125 µg/mL) resulted in decreased attachment and increased appearance of dark cellular remnants of dead cells.

### 3.2. Effect of V. opulus on Intracellular ATP Level, Mitochondrial Membrane Potential, and Apoptosis Induction

**Figure 3:**
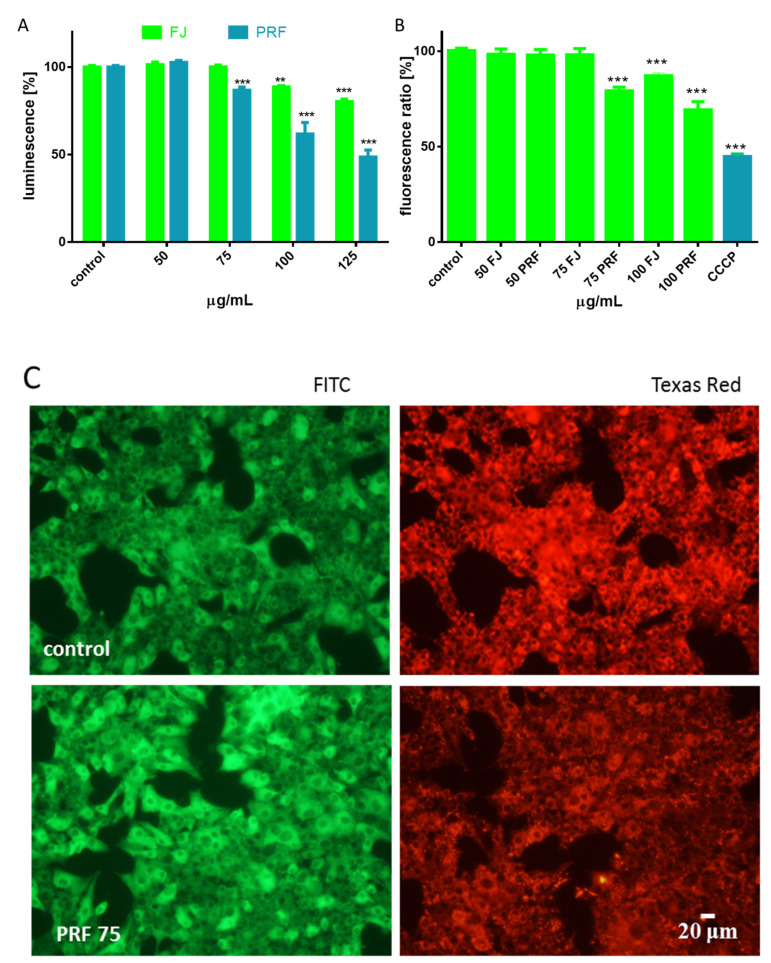
The influence of *V. opulus* extracts on the MIN6 cell ATP level determined by ATP luminescent assay after 24 h exposure of FJ and PRF (**A**); mitochondrial membrane potential was determined with JC-1 probe (**B**), where a positive depolarization control carbonyl cyanide m-chlorophenyl hydrazine (CCCP) was used (50 µM); control cells were not exposed to any compound but the vehicle; values are means ± standard deviations from at least three independent experiments, *n* ≥ 9; statistical significance was calculated versus control cells (untreated), ** *p* ≤ 0.01, *** *p* ≤ 0.001; fluorescent images of MIN6 cells stained with JC-1, where randomly chosen fields were photographed at × 200 magnification with FITC and Texas Red filters (**C**).

To obtain a better understanding of the molecular mechanism of guelder rose cytotoxicity against MIN6 cells, further analyses were performed with extract concentrations higher that IC_0_, but not exceeding IC50 values. Experimental data presented in Figure 3A confirmed that both extracts influenced cellular ATP production. After cell treatment with the phenolics-rich fraction at 75 µg/mL, the ATP level decreased by almost 10–15%, and that effect deepened to 50% at higher concentration (125 µg/mL). FJ reduced the luminescence value by 10–15% at 100–125 µg/mL dosages. Given the fact that the driving force for cellular ATP synthesis is mitochondria, we checked extract influence on the MMP with a JC-1 probe. Our experiments showed that both extracts reduced MMP in a concentration-dependent manner (Figure 3B). While the FJ extract diminished the potential by up to 15%, the phenolics fraction decreased the potential from 20 to 30%. Microscopy images of MIN6 cells (Figure 3C) show that after incubation with PRF (75 µg/mL), the intensity of red fluorescence from punctuate red mitochondrial J-aggregates was lower than that in untreated cells. Simultaneously, enhanced green fluorescence from diffuse green J-monomers was observed in cells with lowered MMP after PRF treatment, thus in unhealthy or dying cells. 

**Figure 4:**
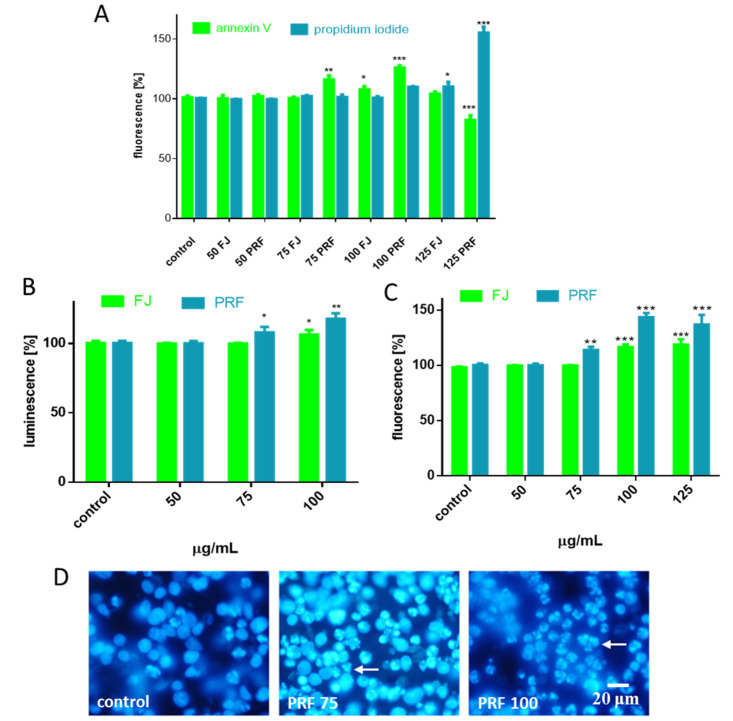
The influence of 24 h of exposure of FJ and PRF on phosphatidylserine externalization on the outer membrane leaflet of apoptotic MIN6 cells and membrane permeabilization detected with Annexin-V-FITC assay kit and propidium iodide staining (**A**); extract influence on caspase-9 activation (**B**) and caspases-3/7 activation (**C**); DAPI-stained nuclei and chromatin condensation observed using fluorescence microscope (Olympus CKX41, Japan), 400 × magnification (**D**). Control cells were not exposed to any compound but the vehicle; values are means ± standard deviations from at least three independent experiments, *n* ≥ 9; statistical significance was calculated versus control cells (untreated), * *p* ≤ 0.05, ** *p* ≤ 0.01, *** *p* ≤ 0.001.

Mitochondria and ATP are considered to be closely related to cellular viability and metabolic activity; thus, due to depletion of ATP and decrease of MMP, we wanted to examine how increasing the concentration of preparations affected the type of cellular death induction. Therefore, we analyzed the proapoptotic influence of preparations by detecting Annexin V bound to the phosphatidylserine externalized on the cell membrane. The highest number of FITC-positive cells was observed for 75–100 µg/mL dosages of PRF (about 15–25%) (Figure 4A). Externalization of phosphatidylserine (PS) was observed for juice at a 100 µg/mL concentration. Otherwise, necrotic membrane integrity damage allows propidium iodide (PI) to enter inside the cells and stain nuclei. A prominent number of necrotic cells (about 50%) was detected for PRF at 125 µg/mL. In the case of juice, slight enhancement of fluorescence for PI-stained nuclei (about 10%) was detected for the highest studied concentration. Taking the obtained data and microscopic observations together (Figure 2), we suppose that the observed elevation of PI fluorescence resulted from secondary necrosis of apoptotic bodies, which were not engulfed by neighboring cells under in vitro experiments.

Apoptotic programmed cell death induction is used in cancer prevention since it is implicated in removal of defective or unwanted cells without leakage of intracellular components and inflammation induction [27]. Critical enzymes involved in apoptosis are caspases; thus, we tested if acute influence of guelder rose extracts leads to activation of these proteinases. Given that PRF decreased the MMP, we tested if it affected initiator caspase-9 activation. As shown in Figure 4B, the PRF at concentrations higher than IC_0_ increased by 10–25% luminescence level of product generated by active caspase-9 (about 10–20%). It is known that caspase-9 is activated by apoptosome, which is a multimeric complex formed by cytoplasmatic Apaf-1 and mitochondrial-associated proteins—Bak, Bax, and cytochrome c—released as a result of mitochondrial membrane depolarization [28]. The primary function of the apoptosome with active caspase-9 is processing effector and executioner caspases-3/7. Once activated, caspases-3/7 cleave many substrates creating hallmarks of apoptosis, such as phosphatidylserine exposure on the outer cellular membrane, cleaved poly (ADP-ribose) polymerase (PARP), chromatin condensation, or genomic DNA fragmentation [28]. Indeed, in our studies significant elevation of fluorescence generated from the Z-DEVD-R110 substrate catalyzed by caspases-3/7 was observed, and it almost exceeded values obtained for control cells by 50% (Figure 4C). Current results are consistent with microscopic observations of nuclear morphology with DAPI staining, where chromatin condensation and nuclear fragmentation after cell incubation with PRF are noticeable (Figure 4D). Based on the above findings, we can conclude that *V. opulus* phenolics induced apoptosis in MIN6 cells through the intrinsic mitochondrial pathway, resulting in initiation of caspase-9 and downstream caspases-3/7 activity. The observed biological activity is in agreement with our previous research, where extracts enriched in phenolic compounds obtained from *V. opulus* fruit induced apoptosis in Caco-2 cells [5]. Taking into account the composition of studied extract, it was shown that chlorogenic acid, the main identified phenolic constituent, and its microbial metabolites induced S-phase cell-cycle arrest and activated caspase-3 at 500–1000 µM concentrations in Caco-2 cells [29]. Recently, it was demonstrated that methanolic extract from *V. grandiflorum* induced apoptosis in lung cancer H1650 and H1299 cell lines [8]. The study revealed the molecular mechanism involved MMP loss, release of cytochrome c into the cytosol, reduction of the anti-apoptotic Mcl-1 protein level, and activation of caspase-3. Thus, *V. opulus* phenolics at higher concentrations can be potentially used as a preventive agent in the human diet against pancreatic cancer development. However, this hypothesis is based on the results obtained in in vitro studies, so it requires more detailed research. 

### 3.3. V. opulus as a Cytoprotective Agent 

**Figure 5:**
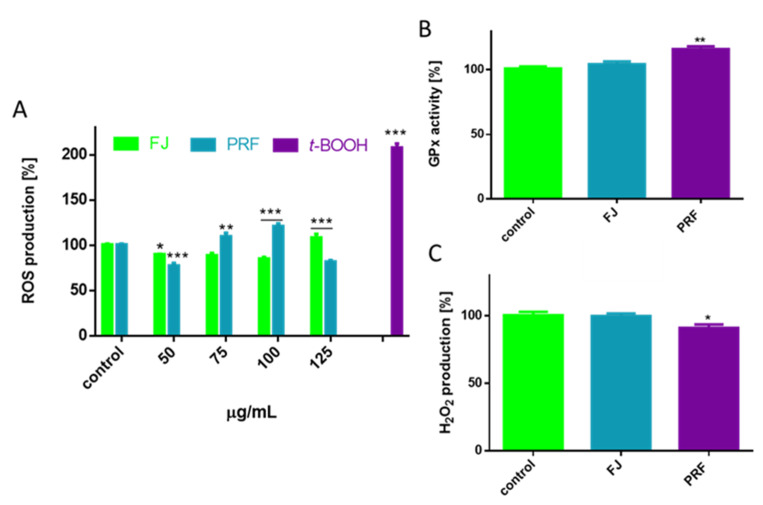
The influence of *V. opulus* extracts on intracellular ROS generation analyzed by DCFH-DA assay after 24 h incubation of MIN6 cells with FJ and PRF (**A**), GPx activity (**B**) and hydroperoxide production level (**C**). Control cells were not exposed to any compound but the vehicle; values are means ± standard deviations from at least three independent experiments, *n* ≥ 9; statistical significance was calculated versus control cells (untreated), * *p* ≤ 0.05, ** *p* ≤ 0.01, *** *p* ≤ 0.001.

ROS are known to act as subcellular messengers; however, their excessive production leads to cell dysfunction, metabolic failure, or even cellular death [30]. Chronic elevation of ROS and excessive oxidative stress very often are the result of increased concentrations of glucose or free fatty acids in the case of type 2 diabetes or obesity [31]. Because polyphenolic compounds possess antioxidant properties allowing the removal and formation of increased ROS, we took an approach to determine the influence of *V. opulus* extracts on intracellular oxidative stress in MIN6 cells. Cell pre-incubation with extracts at IC_0_ concentration decreased intracellular ROS level by 10–20% compared to the cells treated with the vehicle only (Figure 5A); however, the phenolics-rich fraction was more efficient as an oxidative stress reducer. 

Moreover, despite the radical-scavenging activity of polyphenols, the observed cytoprotection may be associated with activation of antioxidant enzymes by phytocompounds. One of the primary antioxidant intracellular enzymes converting hydrogen peroxide into water, or lipid peroxides to their corresponding alcohols, is glutathione peroxidase (GPx) [32]. This enzyme plays crucial role in inhibiting the increase in ROS and lipid peroxidation process; therefore, we analyzed the influence of *V. opulus* at IC_0_ dosages on GPx activity. For this, we performed an indirect assay, which takes advantage of glutathione disulfide formed by the enzymatic action of GPx regenerated by glutathione reductase and can be monitored quantitatively by disappearance of the NADPH co-substrate. The results in Figure 5B show that from both extracts, PRF afforded a significant effect against increasing GPx enzymatic activity by almost 15%. Observed enzymatic activity was further confirmed in measurement of hydrogen peroxide level with assay based on the formation of colored ferric-xylenol orange complex. As demonstrated in Figure 5C cells incubation with PRF decreased hydrogen peroxide production by almost 7%. Because one of the unique features of β-cells is their relatively low expression of ROS-detoxifying antioxidant enzymes [33], the obtained results show *V. opulus* phenolics have a biological impact on the cellular enzymatic antioxidant system. Though, taking into account that reduced glutathione (GSH) is the main cellular antioxidant providing reducing equivalents for GPx catalyzed reduction reaction, more detailed studies including measurements of GSH and oxidized glutathione (GSSG) concentrations, as well as glutathione reductase (GR) activity, should be established.

On the other hand, after cell incubation with extracts at dosages higher than non-cytotoxic, we found an increase in intracellular ROS level: PRF was the strongest oxidative stress inducer, which at 100 µg/mL concentration elevated fluorescence by nearly 25% in comparison to untreated cells. It is known that increase of intracellular ROS level, as a result of mitochondrial depolarization, promotes cytochrome c release from mitochondria into the cytoplasm and induces apoptosis [34]. These data are consistent with our previous finding, where extract concentrations close to the calculated IC_50_ reduced not only intracellular ATP level, but activated caspase-9 and downstream caspases-3/7 inducing the apoptosis program in MIN6. The observed decrease of fluorescence for 125 µg/mL PRF confirms its potent cytotoxicity, inhibition of metabolic activity, cellular damage, and death. 

**Figure 6:**
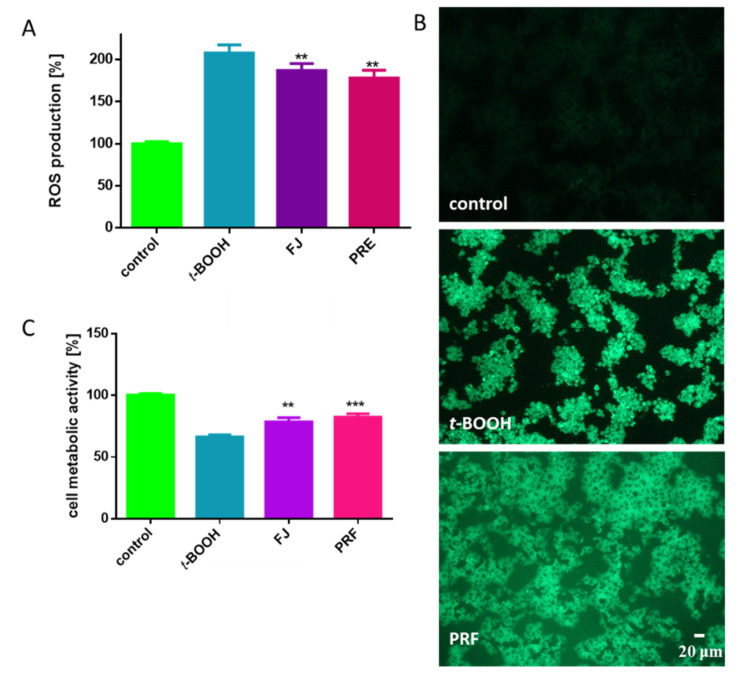
The cytoprotective properties of *V. opulus* extracts at IC_0_ dosages (24 h pre-incubation) against chemical induction (500 µM *t*-BOOH, 2 h) and oxidative stress analyzed by DCFH-DA on MIN6 cells (**A**); cells visualized under fluorescent microscope: low fluorescence in control cells, very high fluorescence in cells after incubation with 500 µM *t*-BOOH, and decreased in cells pre-treated with PRF for 24 h before *t*-BOOH addition (200 × magnification) (**B**); MIN6 metabolic activity analyzed with PrestoBlue assay (**C**). Control cells were not exposed to any compound but the vehicle; values are means ± standard deviations from at least three independent experiments, *n* ≥ 12; statistical significance was calculated versus cells treated with *t*-BOOH, ** *p* ≤ 0.01, *** *p* ≤ 0.001.

The abovementioned low level of antioxidant defense in pancreatic β-cells suggests a need to search for compounds susceptible to protect cells against oxidative damage. To evaluate the cytoprotective properties of *V. opulus* fruit phenolics, we treated cells with *t*-BOOH. It is known in vitro oxidative stress inducer generating radicals leading to alteration of intracellular calcium homeostasis as a result of lipid peroxidation and DNA strand breaks [12,34]. As compared to the untreated cells, *t*-BOOH elevated intracellular ROS production more than twice (Figure 6A). However, toxic effect diminished by nearly 20–30% after cells were pre-incubated with IC_0_ dosages before *t*-BOOH treatment. That quantitative result has been confirmed by fluorescent microscopic observations where cells stained with DCFH-DA fluorogenic dye revealed higher fluorescence (DCF-DA is oxidized by ROS into 2,7-dichlorofluorescein) after *t*-BOOH treatment than cells pre-incubated with PRF (Figure 6B). *t*-BOOH also influenced metabolic activity of MIN6 and lowered it to almost 65% (Figure 6C), and again cell pre-incubation with guelder rose attenuated the toxic effect of *t*-BOOH on metabolic activity by nearly 10–15%. The cytoprotective potential of *V. opulus* fruit phenolics against oxidative damage induced by *t*-BOOH in human adenocarcinoma Caco-2 and mice pancreatic βTC3 cells was demonstrated by us previously [5,20]. Furthermore, extracts were able to protect Caco-2 cells against DNA damage by selected mutagens (methylnitronitrosoguanidine (MNNG) or hydrogen peroxide (H_2_O_2_)) through the induction of DNA repair. There are data demonstrating the protective activity of ethanolic extracts obtained from *V. odoratissimum* dry leaves against hydrogen peroxide-induced oxidative stress in human neuroblastoma SH-SY5Y, which manifests in apoptosis inhibition via decrease of caspase-3 activation and cleavage of PARP, as well as an increase of Bax, a pro-apoptotic protein [14]. It is worth mentioning that the IC_0_ concentrations used in this work (50–75 µg/mL) can be achieved in the gut under physiological conditions, thus *V. opulus* extracts can be effective as cytoprotectants [14]. Studies performed on rats showed that water extracts obtained from *V. opulus* dried leaves had a slight hepatoprotective effect on carbon tetrachloride-induced acute liver toxicity [35]. 

### 3.4. V. opulus Phenolics as Modulators of Fatty Acids Uptake

**Figure 7:**
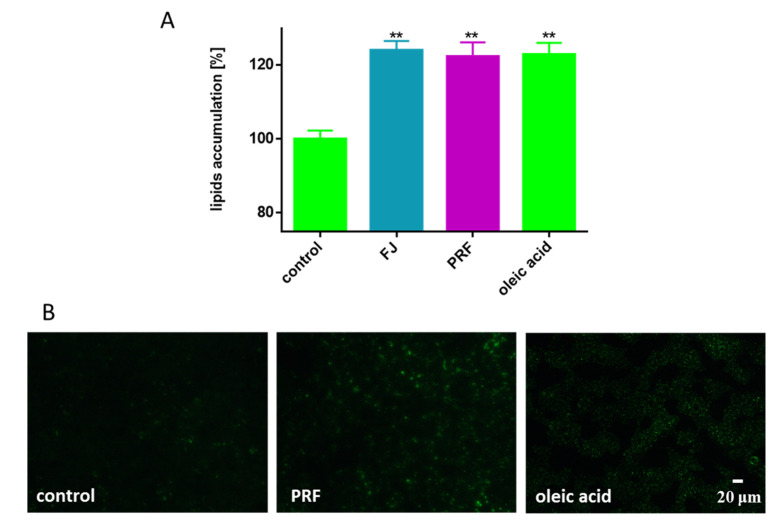
The influence *V. opulus* extracts on the accumulation of lipid droplets in MIN6 cells stained with Nile red observed after treating cells with the extracts at IC_0_ for 24 h (**A**); as a positive control, 100 μM oleic acid was used; control cells were not exposed to any compound but the vehicle; values are means ± standard deviations from at least three independent experiments, *n* ≥ 12; statistical significance was calculated versus control cells ** *p* ≤ 0.01. Cells visualized under fluorescent microscope (200 × magnification) (**B**).

There is growing evidence that β-cell dysfunction, and therefore insulin secretion impairment and cell damage, is associated with amyloid deposits in pancreatic islet cells, oxidative stress, limited incretin action, and increased fatty acids within the pancreas [36]. The disturbance to pancreas function and lipid metabolism very often accompanies diet-related diseases such as overweight, obesity, and type 2 diabetes [31]. Therefore, we decided to investigate the influence of *V. opulus* at IC_0_ concentrations on the level of lipid accumulation by cells co-incubated in the presence of oleic acid (100 µM), which alone was not toxic to the cells after 24 h culture. Oleic acid incubation enhanced lipid accumulation by MIN6 cells by 25%. Taking into account the previously observed inhibition of lipid accumulation in Caco-2 cells by *V. opulus* fruit of phenolics, we suspected to obtain comparable results [5]. Surprisingly, it was found that guelder rose lacked the ability to decrease cellular lipid accumulation in MIN6 cells, and what is more, even slight stimulation of that process was observed (Figure 7A). The influence of preparations on the formation of intracellular lipid droplets stained with Nile red, a hydrophobic fluorescent dye that accumulates in lipid droplets, was observed under microscope. 

**Figure 8:**
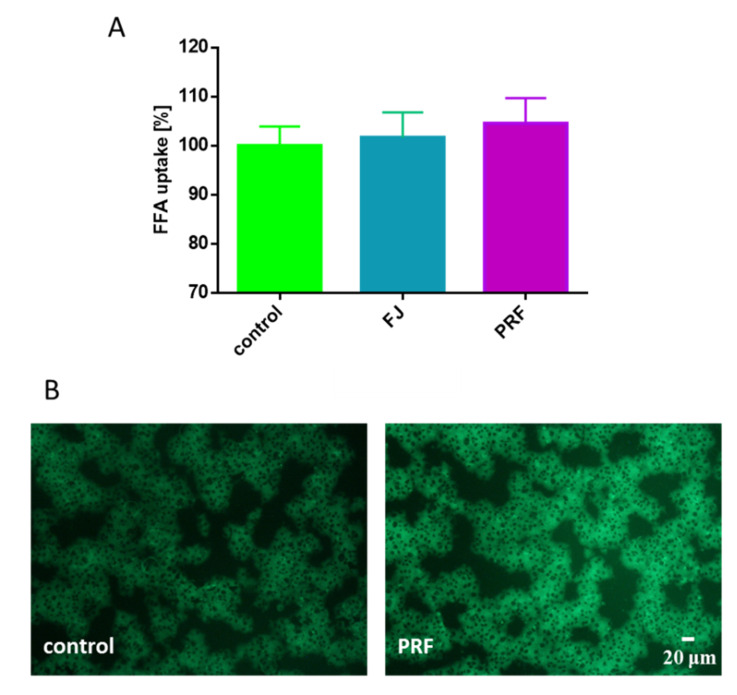
The influence *V. opulus* extracts on fatty acid analogue TF2-C12 uptake measured in MIN6 cells with an FFA uptake assay observed after treating cells with the extracts at IC_0_ for 24 h (**A**); control cells were not exposed to any compound but the vehicle; values are means ± standard deviations, *n* ≥ 5; statistical significance was calculated versus control cells. Cells loaded with TF2-C12 visualized under fluorescent microscope (200 × magnification) (**B**).

The appearance of lipid body organelles is connected to metabolic disorders [37,38]. Interestingly, images presented in Figure 7B showed the cellular appearance of various lipid bodies, although with different numbers and sizes depending on the type of compound intended for incubation with cells. Despite analogous levels of quantitative fluorescence measurement, in cells incubated in the presence of oleic acid, many lipid bodies with comparable, small sizes were present, whereas in cells treated simultaneously with PRF and oleic acid a lower number of lipid droplets was generated, but with greater dimensions. To examine the extracts’ influence on cellular free fatty acid (FFA) uptake, the level of incorporated fluorescent free fatty acid analogue TF2-C12 was measured. As indicated in Figure 8A, the presence of guelder rose preparations insignificantly effected analogue uptake, where increase in fluorescence (circa 5%) was observed in the case of PRF. Microscopic observations revealed that after its uptake, the fluorescent analogue was present mainly in the cytoplasm; however, a small number of spots with intense fluorescence was observed in cells treated with PRF (Figure 8B).

We can suspect the PRF extract influenced the mechanism of fat vesicle creation and lipid droplets transport; however, the proper mechanism, i.e., activity of enzymes involved in lipid metabolism or transport, needs further study. Previously, we showed that phenolics from *V. opulus* reduced the dimensions and number of accumulated lipid droplets on Caco-2 cells [5]. We found the observed mechanism resulted from decreased expression levels of CD36/FAT protein, which is one of the main proteins involved in transmembrane movement of fatty acids, without influence on the expression of the gene encoding PPARα (one of the main cellular regulators of lipid and carbohydrate metabolism). Thus, it can be hypothesized that the observed *V. opulus* phytocompound activity is strongly influenced by the cellular type used as biological model during in vitro studies. We hypothesize that guelder rose may intensify the lipotoxic effects of FFA excess in β-cells generating their dysfunction and death [38]. However, that assumption needs to be verified with more detailed studies including extract influence on oxidative stress or inflammation in the presence of elevated levels of fatty acids. Also, the type of fatty acid present in the cellular medium is important. Studies performed on human 1.1B4 β cells revealed that high lipotoxicity of palmitic acid results from its activity as a generator of intracellular ROS, mitochondrial disruptor, and endoplasmic reticulum stress inducer; on the contrary, oleic acid promoted neutral lipid accumulation and insulin secretion [38]. Comparable results were obtained for rat insulin-producing RINm5F and INS-1E cells [39]. Furthermore, chlorogenic acid, the main constituent of *V. opulus* extracts, attenuated lipotoxicity induced by palmitic acid, reversing oxidative stress and mitochondrial biogenesis dysfunction with activation of SIRT1 in mouse (AML)-12 hepatocytes, as well as prevented endoplasmic reticulum stress-mediated apoptosis in primary rat hepatocytes [40,41]. 

### 3.5. V. opuluce Influence on Insulin and GLP-1 Secretion, and DPP4 Acivity 

**Figure 9:**
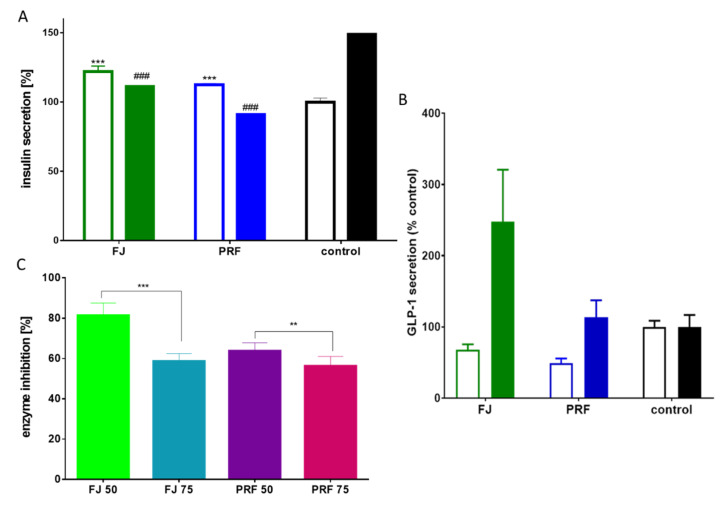
The influence of *V. opulus* extracts on insulin secretion by MIN6 cells (**A**), GLP-1 secretion in GLUTag cells (**B**) in low (open bars) or high (closed bars) glucose conditions. Control cells were not exposed to any compound but the vehicle; values are means ± standard deviations from at least three independent experiments, *n* ≥ 9; statistical significance was calculated versus control cells treated with low glucose *** *p* ≤ 0.001 or high glucose ^###^ *p* ≤ 0.001. Percentage DPP4 enzyme inhibition by FJ and PRF (**C**); values are means ± SD, *n* ≥ 4; statistical significance ** *p* ≤ 0.01, *** *p* ≤ 0.001.

β-cell failure and loss of functionality to secrete enough insulin to maintain glucose homeostasis are critical metabolic disorders contributing to the development of diet-related diseases, such as type 2 diabetes and obesity. Among known cellular models of β-cells, MIN6 cells secrete insulin in response to glucose and other secretagogues agents [21]. Previous reports suggested that chlorogenic acid was able to stimulate insulin secretion from the INS-1E insulin-secreting cell line and rat islets of Langerhans [42]. Taking into account the observed cytoprotective properties of *V. opulus* phenolics, we decided to test their potential as stimulators of glucose-stimulated insulin secretion (GSIS) by MIN6 cells. As presented in Figure 9A, the exposure of MIN6 to elevated 20 mM glucose increased insulin secretion by 50%. Surprisingly, GSIS was significantly reduced by FJ and PRF at IC_0_ dosages, where the phenolic-rich fraction decreased insulin secretion by 40%. Interestingly, at a low-glucose concentration (2 mM) both extracts were able to increase insulin secretion by 15–20%, but the FJ extract was still the stronger activator of that process. There is evidence showing that green tea catechins reveal an inhibitory influence on insulin secretion depending on their structure and concentration, but still are able to decrease hyperglycemia by elevating glucose uptake by cells [43]. The authors have suggested that the inhibitory effect of catechins on GSIS is caused by inhibition of voltage-dependent Ca^2+^ channels, which is mediated partially by membrane hyperpolarization induced by the opening of K^+^ channels. 

Since the secretion of insulin is stimulated after glucagon-like peptide-1 (GLP-1) binding to its GLP1R receptor in the pancreas [18], we decided to study the influence of extracts at IC_0_ dosages on that hormone secretion by GLUTag cells. As shown in Figure 9B, FJ and PRF affected GLP-1 secretion in a differentiated manner dependently on glucose conditions. While supplemented in low-glucose conditions, both FJ and PRF decreased the amounts of secreted GLP-1, as compared to control, by ca. 30% and 50% respectively. Stimulation of GLUTag with FJ in high-glucose conditions resulted in a significant increase in GLP-1 secretion by circa 150%. An insignificant increase was observed in analogous conditions in the case of PRF (circa 15%). Because secreted GLP-1 is extensively hydrolyzed by dipeptidyl peptidase-4 (DPP4), we decided to study the potential of extracts as enzyme activity inhibitors under in vitro conditions. As presented in Figure 9C, both extracts at IC_0_ dosages were able to decrease activity by 40%. Among other phenolic extracts rich in chlorogenic acid, *Xylopia aromatica* was also demonstrated as a DPP4 inhibitor [44]. We can speculate that despite attenuation of GSIS, the pleiotropic properties of *V. opulus* would allow a decrease in postprandial hyperglycemia due to the observed DPP4 inhibition and increase of GLP-1 secretion. 

### 3.6. Membrane Effect of V. opulus

Due to the observed *V. opulus* action on the cellular level, we decided to explore its activities regarding interaction with the cellular membrane. The relevant changes in membrane physical properties strongly influence several cell functions, such as the activity of cell membrane associated enzymes, embedded in membrane lipid bilayer transporters and receptors, and cellular signaling transduction induced by signaling molecules. Based on this, we explored *V. opulus* influence on MIN6 membrane fluidity. It is generally accepted that the fluorophore of Laurdan is mainly located in the phospholipid glycerol backbone and is sensitive to dynamic and free movement of water molecules, which are in the closest proximity to the Laurdan chromophore [45]. Decreased GP values indicate decreased packing arrangement of the membrane and lipid-water interface hydration, which may be related to the increase in membrane fluidity. 

**Figure 10:**
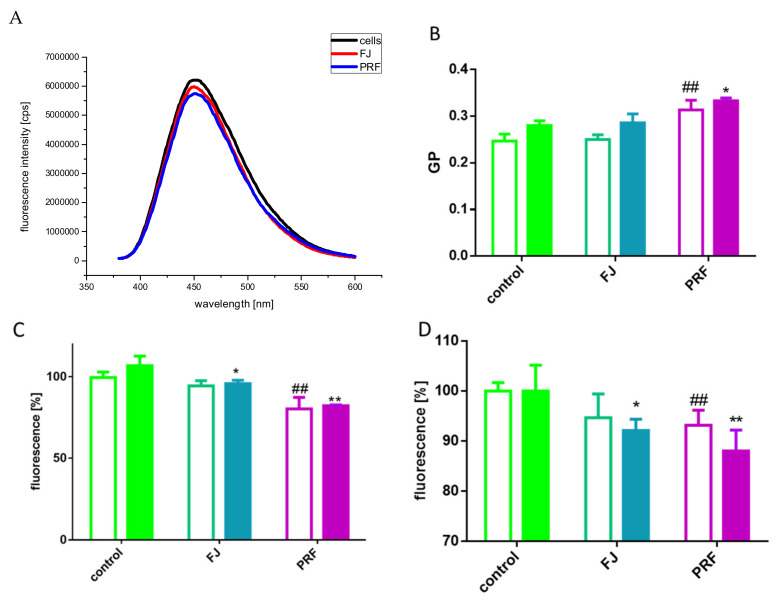
The emission spectra of Laurdan-labeled cells before and after incubation with *V. opulus* extracts at IC_0_ dosages (**A**). Changes in cell membrane fluidity expressed as values of GP for Laurdan probe after cells incubation with extracts in low (open bars) or high (closed bars) glucose conditions (**B**). The influence of extracts on intracellular ROS generation analyzed by DCFH-DA assay in low (open bars) or high (closed bars) glucose conditions (**C**). Hyperpolarizing effect induced by extracts on cellular membrane in low (open bars) or high (closed bars) glucose conditions (**D**). Control cells were not exposed to any compound but the vehicle; values are means ± standard deviations from at least three independent experiments, *n* ≥ 12; statistical significance was calculated versus control cells treated with low glucose * *p* ≤ 0.05, ** *p* ≤ 0.01 or high glucose ^##^ *p* ≤ 0.01.

The emission spectra of cells before and after adding PRF and FJ under standard culture conditions are presented in Figure 10A, and the determined GP values were 0.186, 0.221, and 0.224 for untreated cells, FJ, and PRF, respectively. The increase in GP of the Laurdan probe was observed after incubation with PRF and FJ as compared to that of the control cells. The elevated GP values most probably indicate increased packing density of the lipid polar heads in the membrane, thus decreasing membrane fluidity. Furthermore, the structure of the membrane changes as the glucose or lipid concentration fluctuates [46]. A decrease in membrane fluidity has been documented in diabetic states [47]. 

Taking into account the observed impairment of GSIS, we checked GP parameters for cells cultured in 2 mM and 20 mM glucose concentrations. Elevated glucose concentration not only decreased membrane fluidity, but coincubation with extracts increased the stiffening state (Figure 10B). At the same time, glucose-stimulated elevation of ROS and was attenuated by both preparations (Figure 10C). It was demonstrated that polyphenols protect cellular membranes against oxidation due to membrane stiffening induction, which hinders the spreading of newly generated radicals [4,48]. In that mechanism reactions of polyphenols with nonpolar compounds present in the hydrophobic inner membrane layer are implicated [4]. Nevertheless, polyphenols, via hydrogen bonds, are also involved in shifting fluctuant microdomains known as lipid rafts. Depending on the chemical structure, they cause lipid membrane aggregation and rigidification [49]. On the other hand, we have previously demonstrated the potential of guelder rose to inhibit migration of human MCF7 and HeLa cells [12], and here we can conclude that in the observed mechanism, decreased membrane fluidity is involved. Recent studies clearly show that decreasing fluidity of cell membranes prevents and inhibits metastasis being a viable therapeutic target [50]. Moreover, cellular anchorage to the substrate and to neighboring cells due to the polyphenols interactions with cellular membranes is influenced [34]. Ajdzanovic et al. demonstrated that genistein and daidzein suppressed the metastatic potential of PC cells via increasing membrane stiffness [25]. 

Because compounds lowering intracellular ATP eventually lead to dissipation of plasma membrane potential, in our next step we checked the extract influence on transmembrane potential with a slow-response DiBAC_4_(3) probe that can enter depolarized cells and exhibit enhanced fluorescence [51]. As presented in Figure 10D, *V. opulus* extracts reduced fluorescence emission; thus, hyperpolarized cellular membrane and reduction was more significant in cells cultured in 20 mM glucose. In the classic pathway of insulin secretion after glucose entry into β-cells, a rise in the ATP:ADP ratio closes ATP-dependent K+ channels, which in turn opens the voltage-gated calcium channels, allows Ca^2+^ to enter the cells, and leads to subsequent insulin exocytosis [52]. Previous studies indicated that catechins inhibit GSIS in pancreatic β-cells by decreasing the intracellular Ca^2+^ concentration, which was partially caused by membrane hyperpolarization resulting from the activation of K^+^ channels [43]. Recently, it was demonstrated that epicatechin potentiates GSIS in INS-1 cells through activation of the Ca^2+^/calmodulin-dependent protein kinase (CaMK) II pathway and as a ligand for GPR40 receptor, which in turn is activated during stimulation by GLP-1 [15]. Taking these into account, we suspect that GSIS inhibition by *V. opulus* phenolics is partly due to polyphenol-membrane interactions leading to the impairment of signal transduction and membrane hyperpolarization, although further studies are needed to support this hypothesis.

**Figure 11:**
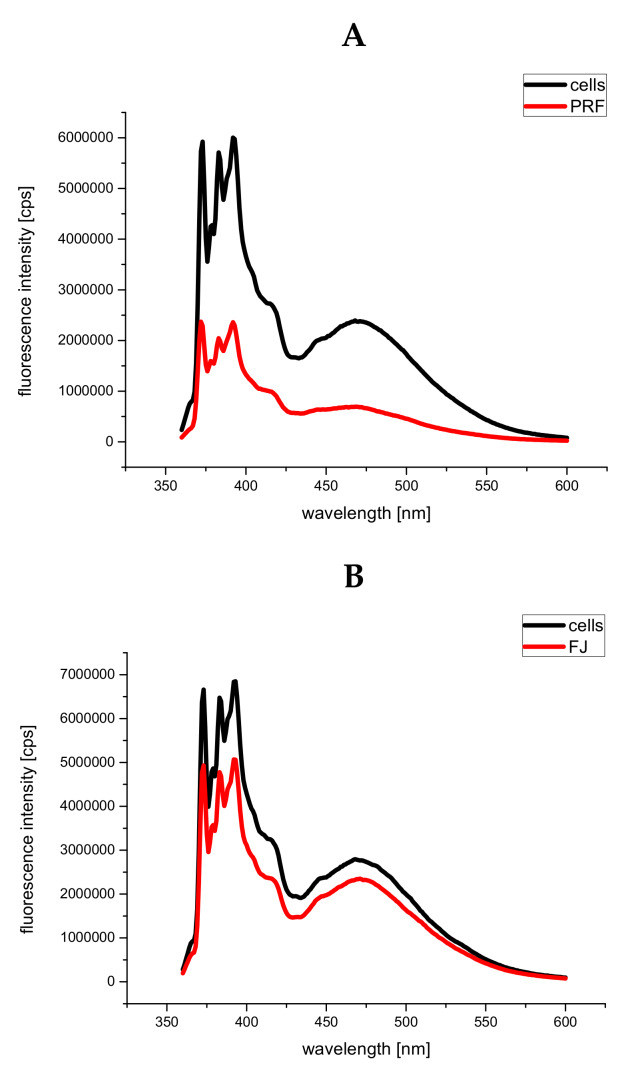
The emission of pyrene-labeled cells before and after incubation with *V. opulus* PRF (**A**) and FJ (**B**) extracts at IC_0_ dosages; presented data are representative emission spectra of pyrene-labeled cells before and after incubation with PRF and FJ, *n* = 3.

Next, we evaluated *V. opulus* extracts on membrane viscosity at the level of the fatty acid chains in the phospholipid bilayers, and thus lateral phase separation, which was estimated based on the ratio of excimer/monomer fluorescence of pyrene. For the excimer to be formed, it is essential that the membrane is in the fluid state. Excimer formation requires close contact between an excited molecule of pyrene with another non-excited pyrene molecule. The ratio of excimer/monomer fluorescence intensity can therefore be used as an index of lateral diffusion (lateral mobility of the membrane environment); thus, the higher the ratio, the higher the membrane fluidity [53]. The emission spectra of the cells labeled with pyrene are shown in Figure 11A,B. The determined values of the ratio of excimer/monomer fluorescence intensity I_470_/I_396_ are gathered in Table 2. The lower value of I_470_/I_396_ obtained for cells incubated with PRF and FJ, as compared to the control cells, may indicate decreased membrane fluidity in cells cultured under standard glucose conditions.

### 3.7. Human Serum Albumin Fluorescence Quenching by PRF

One of the crucial factors that determines the bioavailability and safety properties of a potential drug is the ability of the drug to bind to HSA [54]. Therefore, we have undertaken efforts to examine the interactions between HSA and biologically active *V. opulus* PRF. For this purpose, we have applied the standard method based on the fluorescence quenching of single tryptophan containing HSA. This method has been previously used by other authors [55], and it was useful in providing information concerning the affinity of polyphenolic compounds to that protein. 

**Figure 12:**
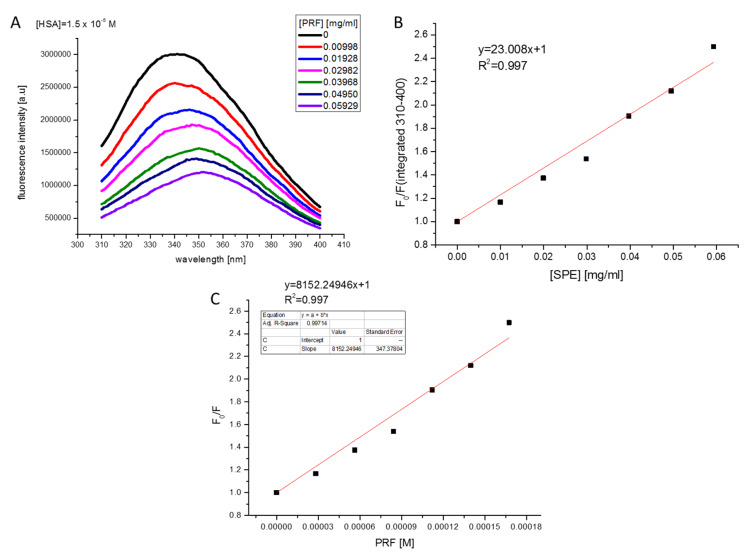
Fluorescence spectrum of HSA (1.5 × 10^−5^ M, excitation at 295 nm) and its changes upon increasing concentrations of the PRF extract (**A**). Stern–Volmer plot for HSA quenching by PRF (concentration of PRF in mg/mL) (**B**). Stern-Volmer plot for HSA quenching by PRF (concentration of PRF in expressed as chlorogenic acid equivalent [M]) (**C**).

Fluorescence spectrum of HSA and its changes upon increasing concentrations of PRF are presented in Figure 12A–C together with the Stern-Volmer plot. As it can be seen, addition of the PRF component to HSA solution resulted in tryptophan fluorescence quenching of the albumin. Moreover, a slight red shift of the HSA emission spectra maximum may be observed. Due to the presence of this alteration to the fluorescence maximum, the Stern-Volmer plot has been obtained from the integrated fluorescence intensities (the area under the spectrum with the wavelength range of 310–400 nm). 

The determined value of the Stern–Volmer constant was (8.152 ± 0.347) × 10^3^/M and was lower as compared to the value of 4.23 × 10^4^/M, previously determined for HSA quenching by 3,4-dimethoxycinnamic, which belongs to the coffee polyphenols family [55]. The time-resolved fluorescence measurements (results not shown) have indicated that the lifetime of HSA (average lifetime of around 6 ns) has not changed significantly upon adding PRF in the tested concentration range, which may indicate that PRF quenches the tryptophan fluorescence of HSA via a static quenching mechanism. The bimolecular quenching constant of tryptophan HSA fluorescence by PRF is determined from the following equation:

(3) $$\frac{F_{0}}{F}=1+k_{q}\cdot \tau_{0}\left[Q\right]=1+K_{SV}\left[Q\right]$$

and it is equal to

(4) $$\;k_{q}=\frac{K_{SV}}{6\times 0.000000001}=1.36\times 10^{12}\;M^{-1}\cdot s^{-1}$$

This value is above the diffusion-limited rate constant of the biomolecule equal to 1 × 10^10^ M^−1^∙s^−1^, which suggests the presence of binding interactions of the compounds studied with HSA and most probably indicates involvement of static quenching mechanism. The static mechanism of HSA fluorescence quenching by polyphenolic compounds from *Chaenomeles japonica* has also been previously proposed by other authors [56]. In the studied extract, large quantities of (−)-epicatechin, chlorogenic acid, monomers, and polymers of procyanidins were identified. 

## 4. Conclusions

**Figure 13:**
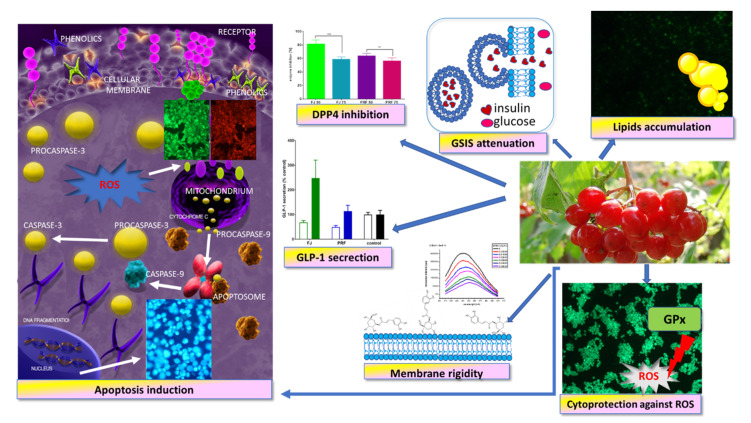
*Viburnum opulus* phenolic compounds as cytoprotective agents influencing MIN6 cell biological activity—proposed mechanism of action. They possess cytoprotective activity against ROS generation, induce apoptotic-type cell death, stimulate GLP-1 secretion, and inhibit DPP4 activity; however, they attenuate GSIS and enhance lipid accumulation.

According to the World Health Organization, there is a growing population of people suffering from diet-related diseases, such as type 2 diabetes or obesity. Recent clinical studies demonstrated that pre-diabetes or pre-obesity can be delayed with lifestyle improvement, mainly through physical activity and a diet enriched with bioactive compounds that can affect cellular metabolism. There is growing evidence that components of different parts of plants, including fruit, have beneficial effects in fighting metabolic diseases [57,58]. Taking into account that the pancreas is involved in nutrient metabolism regulation and glucose homeostasis, we determined the influence of *Viburnum opulus* on the β-cell MIN6 line. In this study, we revealed that the phenolic-rich fraction obtained from fruit juice (PRF) had stronger than the FJ antioxidant properties with GPx activation, and it possessed cytoprotective activities against *t*-BOOH-induced intracellular oxidative stress (Figure 13). Because one of the unique features of β-cells is their relatively low intracellular ROS-detoxifying system, the obtained results show *V. opulus* phenolics have a biological impact on oxidative stress defense. Moreover, elevated extract concentrations induced cell apoptosis through the intrinsic mitochondrial pathway, resulting in initiation of caspase-9 and downstream caspases-3/7 activation. We want to emphasize that apoptotic-type cell death is less destructive than necrosis to neighboring cells, owing to the lack of active enzymes or inflammatory signals released from dying cells.

As demonstrated, polyphenolic rich fraction obtained from FJ revealed stronger activity than juice. These results indicate that both extracts can cause a concentration-dependent influence on cellular activities according to the content of phenolic compounds. PRF fraction possessed concentration of phenolic compounds almost 80-fold higher than FJ. Probably, the most responsible for the described activities is chlorogenic acid, quantitatively the main component of both extracts. Still, comparing concentrations of cytoprotective properties and the concentration of cytotoxicity demonstrated that to decrease MIN6 cell viability to 50%, the IC_0_ dose of FJ (180 µg/mL) needs to be doubled, and the IC_0_ concentration almost tripled in the case of PRF (135 µg/mL). With reference to the amount of phenolic compound, the PRF extract should be expected as more biologically active than FJ, but observed impact is not so distinctive. FJ potential may be associated with the presence of other phenolic compounds in its ingredients, which in turn were lost during solid-phase extraction and were not detected in PRF. Furthermore, in FJ, besides phenolic compounds there are other ingredients (i.e., sugars, proteins, organic acids, minerals) that, through chemical interactions and synergistic effects between the components, may have a stronger influence on the observed activity. It is worth mentioning that the IC_0_ concentrations used in this work (50–75 µg/mL) can be achieved in the gut under physiological conditions, thus *V. opulus* extracts can be effective in cellular metabolism regulating food supplement components. The results of the HSA fluorescence-quenching studies indicate that the PRF extract has binding affinity to human serum albumin, which is one of the factors that determine drug bioavailability.

Still, due to the relatively small differences between cytoprotective and cytotoxic concentrations, it can be assumed that the extract doses used may cause cellular damage instead of cytoprotection. Indeed, we have demonstrated that *V. opulus* extracts may induce MIN6 cells functional failure at IC_0_ dosages through the decrease of GSIS. We supposed that GSIS inhibition resulted from extract component modulation of the cellular membrane dynamic. We hypothesize that guelder rose phenolics decreased the membrane fluidity at elevated glucose concentrations and down-regulated insulin exocytosis via hyperpolarization, leading to decreased intracellular Ca^2+^ concentration. Furthermore, the increased GP values and the lower values of excimer/monomer fluorescence intensity suggest that the extract components affected the membrane microviscosity by causing an increase in viscosity. Based upon our data, it can be assumed that under conditions of postprandial glucose elevation *V. opulus* extract may deepen insulin insufficiency, decrease glucose uptake by peripheral tissues, and elevate hyperglycemia. 

More importantly, we have shown that extracts increased FFA uptake and intracellular accumulation of lipid droplets in insulinoma cells. Increased intracellular triglyceride accumulation intensifies ROS generation leading to a lipotoxic effect through mitochondrial fatty acid oxidation, endoplasmic reticulum stress, and apoptosis induction. This promotes a decrease of β-cell mass, reduction of insulin secretion, and subsequent fatty replacement, leading to pancreatic steatosis [59]. Our previous results showed that guelder rose was able to decrease uptake of free fatty acids by Caco-2 cells [5]. These cells are used as an artificial intestine model during studies of compound uptake and bioavailability, as they maintain part of the functional capacity of epithelium in vitro despite their malignant origin. In that case, *V. opulus* was implicated in the decrease of *CD36/FAT* protein expression, which is one of the main proteins involved in fatty acid transmembrane movement. MIN6 cells are known to express CD36/FAT [60]; hence, we suppose the observed guelder rose activity might be dependent on cellular type. The data also raised a new question, whether extract components triggered other effects in MIN6 cells, such as formation of toxic ceramide species, altered lipid metabolism, different FFA receptor signaling, or lipid droplet protein engagement [61,62]. 

In regulating glucose levels, besides insulin, the GLP-1 protein is involved. GLP-1 stimulates insulin secretion after binding to its GLP1R receptor in the pancreas; thus, insulin and GLP-1 effects are physiologically intertwined. Under physiological conditions GLP-1 is extensively hydrolyzed by DPP4, which significantly limits insulin secretion by β-cells. Our study has clearly shown other important properties of *V. opulus* extracts: they stimulated GLP-1 secretion by GLUTag cells in the presence of an elevated glucose concentration, and they inhibited DPP4. We can speculate that despite attenuation of GSIS, the multifunctional properties of *V. opulus* still would be able to decrease, at least partially, postprandial hyperglycemia regardless of insufficient or inappropriate insulin secretion. Because one limitation of our study was estimating the inhibitory activity of extracts on DPP4 isolated enzymes, it will be interesting to determine whether inhibition of cellular DPP4 will be confirmed. Thus, we cannot exclude the possibility that *V. opulus* lipotoxic effects against pancreatic cells would be stronger than its effect on extending the GLP-1 protein half-life. 

Taken together, despite the cytoprotective activity against generated intracellular oxidative stress, *V. opulus* revealed potential lipotoxic effects, as well as decreased insulin secretion, from MIN6 cells. These findings are relevant in understanding *V. opulus* limitations in the development of diet supplement components, especially designed for the prevention and treatment of postprandial glucose elevation. Still, more detailed studies of guelder rose fruit extracts effects on insulinoma cells are required, particularly after in vitro digestion, treatment, or incubation with gut microflora, which may lead to changes in the composition of the studied chemicals. 

## Figures and Tables

**Table 1:** Individual phenolic compounds in *Viburnum opulus* fruit samples [5].

Phenolic Compounds	FJ	PRF
	mg/g of Juice	mg/g of Extract
Flavanols	1.21 ± 0.01	100.74 ± 0.10
Procyanidin B1	0.31 ± 0.00	29.76 ± 0.05
(+)-Catechin	0.68 ± 0.01	53.18 ± 0.03
Procyanidin B2	0.22 ± 0.00	17.79 ± 0.02
Hydroxycinnamic Acids (HCA)	7.97 ± 0.02	645.85 ± 2.00
Neochlorogenic acid	n.d.	0.36 ± 0.00
Chlorogenic acid	7.97 ± 0.02	645.49 ± 2.00
Flavonols	0.01 ± 0.00	2.35 ± 0.00
Rutin	0.01 ± 0.00	1.09 ± 0.00
Isorhamnetin	0.002 ± 0.000	0.12 ± 0.00
Isorhamnetin 3-*O*-rutinoside	0.01 ± 0.00	0.84 ± 0.00
Quercetin	n.d.	0.30 ± 0.00
Anthocyanins	1.12 ± 0.00	78.06 ± 0.68
Cyanidin-3-*O*-sambubioside	0.85 ± 0.00	55.78 ± 0.69
Cyanidin-3-*O*-glucoside	0.20 ± 0.01	17.08 ± 0.01
Cyanidin-3-*O*-rutinoside	0.06 ± 0.00	5.21 ± 0.01
Phenolics Total	10.32 ± 0.03	827.00 ± 1.68

FJ—fresh juice, PRF—phenolic rich fraction from FJ, n.d., not detected.

**Table 2:** The determined values of the ratio of excimer/monomer fluorescence intensity I_470_/I_396._

Samples	I_470_/I_396_
cells	0.514 ± 0.010
cells + FJ	0.408 ± 0.016
cells	0.508 ± 0.015
cells + PRF	0.441 ± 0.022

means ± standard deviations from three independent experiments, *n* = 3.
